# Supplementary material for: Meta-Analysis and Gene Set Enrichment Relative to ER Status Reveal Elevated Activity of MYC and E2F in the “Basal” Breast Cancer Subgroup
Source: PLoS One. 2009 Mar 9;4(3):e4710. doi: 10.1371/journal.pone.0004710 (PMC2650420; doi:10.1371/journal.pone.0004710)
Supplement: Dataset S1 — GSEA results from first screen. The file contains a zipped archive of the HTML results of the second GSEA screen. Reports for enriched or depleted datasets may be accessed from the “index.html” link within the my_analysis.GseaPreranked.1219975950625.rpt.zip ZIP archive. Enrichment plots are available for each of the 50 most enriched and depleted gene sets. (13.32 MB ZIP) [file pone.0004710.s001.zip › my_analysis.GseaPreranked.1219975950625.rpt.zip!//CMV_IE86_UP.html]

Details for gene set CMV\_IE86\_UP[GSEA]

|  || Dataset | zSco.ERneg.to.ERpos\_collapsed |
| Phenotype | NoPhenotypeAvailable |
| Upregulated in class | na\_pos |
| GeneSet | CMV\_IE86\_UP |
| Enrichment Score (ES) | 0.6863181 |
| Normalized Enrichment Score (NES) | 2.5207214 |
| Nominal p-value | 0.0 |
| FDR q-value | 0.0 |
| FWER p-Value | 0.0 |
Table: GSEA Results Summary

  

Fig 1: Enrichment plot: CMV\_IE86\_UP      
 Profile of the Running ES Score & Positions of GeneSet Members on the Rank Ordered List

  

| PROBE | GENE SYMBOL | GENE\_TITLE | RANK IN GENE LIST | RANK METRIC SCORE | RUNNING ES | CORE ENRICHMENT || 1 | FOXM1 |  |  | 21 | 6.760 | 0.0392 | Yes |
| 2 | CCNE1 |  |  | 24 | 6.716 | 0.0796 | Yes |
| 3 | MCM5 |  |  | 31 | 6.533 | 0.1185 | Yes |
| 4 | BLM |  |  | 56 | 6.255 | 0.1544 | Yes |
| 5 | NASP |  |  | 59 | 6.201 | 0.1917 | Yes |
| 6 | BTG3 |  |  | 102 | 5.850 | 0.2238 | Yes |
| 7 | RFC3 |  |  | 164 | 5.493 | 0.2524 | Yes |
| 8 | MCM6 |  |  | 198 | 5.317 | 0.2820 | Yes |
| 9 | PTTG1 |  |  | 225 | 5.206 | 0.3114 | Yes |
| 10 | TPX2 |  |  | 231 | 5.180 | 0.3423 | Yes |
| 11 | MCM7 |  |  | 289 | 4.970 | 0.3680 | Yes |
| 12 | EZH2 |  |  | 312 | 4.888 | 0.3958 | Yes |
| 13 | MYBL2 |  |  | 418 | 4.629 | 0.4158 | Yes |
| 14 | MAD2L1 |  |  | 477 | 4.446 | 0.4383 | Yes |
| 15 | MCM2 |  |  | 630 | 4.080 | 0.4514 | Yes |
| 16 | MCM3 |  |  | 656 | 4.019 | 0.4738 | Yes |
| 17 | KIF11 |  |  | 713 | 3.909 | 0.4931 | Yes |
| 18 | RFC2 |  |  | 762 | 3.821 | 0.5126 | Yes |
| 19 | MSH2 |  |  | 819 | 3.748 | 0.5310 | Yes |
| 20 | TYMS |  |  | 829 | 3.738 | 0.5528 | Yes |
| 21 | H2AFX |  |  | 930 | 3.591 | 0.5670 | Yes |
| 22 | PPIH |  |  | 943 | 3.562 | 0.5875 | Yes |
| 23 | FLAD1 |  |  | 1059 | 3.402 | 0.5994 | Yes |
| 24 | UNG |  |  | 1070 | 3.390 | 0.6191 | Yes |
| 25 | NP |  |  | 1241 | 3.199 | 0.6256 | Yes |
| 26 | DNAJC9 |  |  | 1243 | 3.198 | 0.6448 | Yes |
| 27 | CDC2 |  |  | 1259 | 3.182 | 0.6629 | Yes |
| 28 | PCNA |  |  | 1346 | 3.087 | 0.6750 | Yes |
| 29 | RAB27A |  |  | 1438 | 3.011 | 0.6863 | Yes |
| 30 | TFAM |  |  | 1710 | 2.755 | 0.6825 | No |
| 31 | CSPG5 |  |  | 1934 | 2.583 | 0.6813 | No |
| 32 | RPA1 |  |  | 2380 | 2.270 | 0.6615 | No |
| 33 | CDT1 |  |  | 2522 | 2.190 | 0.6641 | No |
| 34 | BARD1 |  |  | 3028 | 1.920 | 0.6376 | No |
| 35 | UMPS |  |  | 3124 | 1.877 | 0.6417 | No |
| 36 | TOPBP1 |  |  | 3475 | 1.722 | 0.6258 | No |
| 37 | NUP214 |  |  | 3486 | 1.716 | 0.6354 | No |
| 38 | HMGB2 |  |  | 3883 | 1.558 | 0.6149 | No |
| 39 | POLA1 |  |  | 3967 | 1.525 | 0.6179 | No |
| 40 | KIAA0101 |  |  | 4770 | 1.230 | 0.5649 | No |
| 41 | RRM1 |  |  | 4872 | 1.197 | 0.5645 | No |
| 42 | DLEU1 |  |  | 4922 | 1.183 | 0.5679 | No |
| 43 | POLD3 |  |  | 6667 | 0.601 | 0.4401 | No |
| 44 | RAD51C |  |  | 8329 | -0.007 | 0.3150 | No |
| 45 | DCK |  |  | 8935 | -0.280 | 0.2711 | No |
| 46 | CASP3 |  |  | 9899 | -0.783 | 0.2033 | No |
| 47 | SLC29A1 |  |  | 10768 | -1.365 | 0.1461 | No |
| 48 | TMEM106C |  |  | 12224 | -2.877 | 0.0539 | No |
| 49 | DDX23 |  |  | 13079 | -4.749 | 0.0182 | No |
Table: GSEA details [plain text format]

  

Fig 2: CMV\_IE86\_UP: Random ES distribution      
 Gene set null distribution of ES for **CMV\_IE86\_UP**
